# *Colletotrichum higginsianum* as a Model for Understanding Host–Pathogen Interactions: A Review

**DOI:** 10.3390/ijms19072142

**Published:** 2018-07-23

**Authors:** Yaqin Yan, Qinfeng Yuan, Jintian Tang, Junbin Huang, Tom Hsiang, Yangdou Wei, Lu Zheng

**Affiliations:** 1 The Key Lab of Plant Pathology of Hubei Province, Huazhong Agricultural University, Wuhan 430070, China; zkyyanyaqin@163.com (Y.Y.); yuanqf1989@163.com (Q.Y.); jintiantang@yeah.net (J.T.); junbinhuang@mail.hzau.edu.cn (J.H.); 2 School of Environmental Sciences, University of Guelph, Guelph, ON N1G 2W1, Canada; thsiang@uoguelph.ca; 3 Department of Biology, University of Saskatchewan, Saskatoon, SK S7N 5E2, Canada; yangdou.wei@usask.ca

**Keywords:** *Arabidopsis*, *Colletotrichum higginsianum*, genomics, hemibiotrophic infection, plant–fungal interactions, virulence factors

## Abstract

*Colletotrichum higginsianum* is a hemibiotrophic ascomycetous fungus that causes economically important anthracnose diseases on numerous monocot and dicot crops worldwide. As a model pathosystem, the *Colletotrichum–Arabidopsis* interaction has the significant advantage that both organisms can be manipulated genetically. The goal of this review is to provide an overview of the system and to point out recent significant studies that update our understanding of the pathogenesis of *C. higginsianum* and resistance mechanisms of *Arabidopsis* against this hemibiotrophic fungus. The genome sequence of *C. higginsianum* has provided insights into how genome structure and pathogen genetic variability has been shaped by transposable elements, and allows systematic approaches to longstanding areas of investigation, including infection structure differentiation and fungal–plant interactions. The *Arabidopsis-Colletotrichum* pathosystem provides an integrated system, with extensive information on the host plant and availability of genomes for both partners, to illustrate many of the important concepts governing fungal–plant interactions, and to serve as an excellent starting point for broad perspectives into issues in plant pathology.

## 1. Introduction

**Figure 1:**
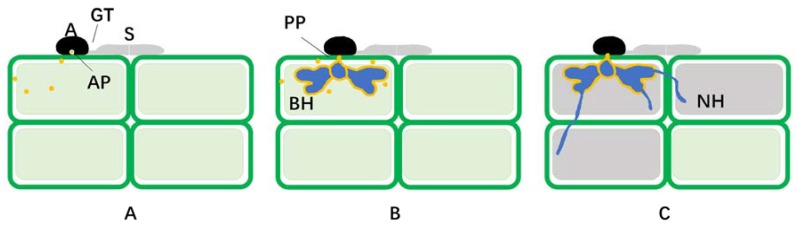
Infection structure development and effector localization in *Colletotrichum higginsianum*. (**A**) Appressorial formation on the leaf surface at 24 hpi. Spores (S) adhere to the host cuticle and produce a germ tube (GT), and an appressorium (**A**) is formed to penetrate plant epidermal cells directly. Effectors, marked with yellow dots, accumulate at the appressorial pore (AP) and then are secreted from the pore; (**B**) the biotrophic infection phase at 40 hpi. A penetration peg (PP) develops from the base of the appressorium and penetrates the host cuticle and cell wall. Primary biotrophic hyphae (BH) develop inside the epidermal cell and invaginate the plant plasma membrane. The host protoplast remains alive during this biotrophic stage of the interaction. Effectors accumulate at the biotrophic interfacial bodies, the yellow layer outside the primary hyphae, and then are secreted to the host cell from the biotrophic interfacial bodies; (**C**) the necrotrophic infection phase at 55 hpi. Secondary necrotrophic hyphae (NH) later develop from the BH and spread into the surrounding cells without biotrophic interfacial bodies and directly penetrate host cytoplasm. The host epidermal cell shaded in dark gray then dies after NH production. All graphics were derived from original micrographs for easier visualization.

*Colletotrichum* is a large ascomycete genus comprising more than 190 species, many of which cause devastating diseases on a large range of agricultural and horticultural crops worldwide [1]. Among species of *Colletotrichum*, *C. higginsianum* is classfied in a main phyogenetic clade within the *C. destructivum* complex, and causes anthracnose disease on a wide range of cruciferous plants, such as species of *Brassica* and *Raphanus* as well as the model plant *Arabidopsis thaliana* [2,3,4]. Since most *A. thaliana* ecotypes are susceptible to *C. higginsianum*, the pathogen can be regarded as adapted for *A. thaliana* [5]. As a typical hemibiotrophic fungus, *C. higginsianum* develops a series of specialized infection structures including germ tubes, appressoria, primary biotrophic hyphae (BH), and secondary necrotrophic hyphae (NH) (Figure 1). Thus, *C. higginsianum* is one of the best-studied species within the genus *Colletotrichum* because of its interesting infection strategy, and the ease with which it can be cultured axenically and transformed with high efficiency by T-DNA transfer mediated by *Agrobacterium tumefaciens*. Furthermore, complete genome sequences and transcriptome data are available [6]. For these reasons, the *C. higginsianum–Arabidopsis* pathosystem has become an attractive model for research on the molecular basis of fungal pathogenicity and plant–fungal interactions.

## 2. Infection Strategies

At the start of the hemibiotrophic life cycle of *C. higginsianum* on *Arabidopsis*, conidia land on the leaf surface and produce germ tubes, which then produce appressoria to penetrate the leaf surface [7]. As they mature, cell walls of appressoria become melanized while suitable solutes will accumulate in the cytoplasm (Figure 1A). High turgor pressure builds up by water diffusion into appressoria, which provides the force for the peg to penetrate through the plant cell wall. Within a breached epidermal cell, the initial narrow hypha from the peg gives rise to a swollen, sac-like BH. The BH enlarge and form lateral bulbous lobes, resembling a haustorium. The fungus establishes itself as a biotroph within 36 h post infection by forming a multiseptate, multilobed structure, variable in shape and confined within the initially infected epidermal cells (Figure 1B). At this stage of the interaction, infected cells can still plasmolyse normally, and the host plasmalemma and tonoplast remained functional [8]. Upon subsequent colonization of neighbouring cells at 72 h post-infection, a switch in both hyphal morphology and trophic relationship occur. At the periphery of the lobed BH, outgrowths develop rapidly to produce narrow NH (Figure 1C). These numerous hyphae radiating from each BH grow through the adjacent cell walls and infect surrounding cells. Narrow NH grow rapidly, and hyphal spread will eventually lead to necrotic lesions with the appearance of water-soaked lesions on the surface of the infected host as soon as 84 h post-infection [9]. In necrotic tissues, acervuli form to produce numerous conidia.

Some *Colletotrichum* species including *C. graminicola*, *C. falcatum*, *C. caudatum* and *C. sublineola* have an intracellular hemibiotrophic infection stage with a short period of biotrophy [10,11,12]. Unlike *C. higginsianum*, the BH of these four species grow not only in the initially infected cell but also into cells adjacent to the first infected cell before the fungi switch to NH, which ramify throughout host cells [13]. Similarly, for infection by another hemibiotrophic fungus *Magnaporthe oryzae*, once the fungus has breached the outer plant surface, it begins an extended period of biotrophic invasion of successive host cells. Rice cells invaded by *M. oryzae* plasmolyze as hyphae colonize them, but plasmolysis stops when the fungus grows into neighboring rice cells. In this form of hemibiotrophy, necrotrophic growth appears to be triggered at four to five days post-inoculation, when macroscopic lesions appear [14]. In contrast, biotrophic infection by *C. higginsianum* is entirely restricted to the first infected epidermal cell, and development then switches to necrotrophic growth, which then spreads within and between host cells, and kills host cells ahead of infection.

## 3. Genomics and Genetics

### 3.1. Genome Sequencing and Assembly

Two largescale genome projects have been completed for *C. higginsianum* aiming to produce high-quality assemblies to provide resources for comparative genomics and molecular analyses of fungal pathogenicity, which allow the identification of genes relevant to each stage of plant infection. In 2012, the first genome of *C. higginsianum* strain IMI 349063 was reported by O’Connell using a multi-source method, including short-read data from 454 GSFLX (350 bp) and Illumina GAII (100 bp) sequencing platforms together with a smaller number of longer Sanger reads. Optical mapping showed that the genome of *C. higginsianum* strain IMI 349063 was 53.4 Mb distributed among 12 chromosomes, including two mini chromosomes less than 1 Mb in size [15]. However, the actual assembly was composed of over 10,000 contigs. This genome is smaller than other sequenced *Colletotrichum* genomes (88.3 Mb, 55.6 Mb and 57.4 Mb such as *C. orbicular*e (88.3 Mb), *C. gloeosporioides* (55.6 Mb) and *C. graminicola* (57.4 Mb) [15,16]. Surprisingly, more genes (16172) were predicted from the *C. higginsianum* assembly in contrast to assemblies of *C. orbiculare* (13479), *C. gloeosporioides* (15469) and *C. graminicola* (12006) [12,16]. One limitation of this assembly was that many of the predicted protein-coding genes were truncated or split between contigs, resulting in multiple gene calls. The fragmented nature of the assembly leading to incomplete gene calls was confirmed by a series of problematic experiments in our labs.

Recently, *C. higginsianum* strain IMI 349063 had been re-sequenced using the single-molecule real-time (SMRT) technique, and combined with previous optical mapping data, has achieved a gapless assembly of all 12 chromosomes except for the ribosomal DNA repeat cluster on chromosome 7 [17]. This assembly of nearly all chromosomes represents the most complete genome assembly to date of any *Colletotrichum* species, and becomes part of a short list of completely assembled genomes of phytopathogenic fungi, namely *Zymoseptoria tritici*, *Sclerotinia sclerotiorum*, *Botrytis cinerea*, *Verticillium dahliae* and *Fusarium graminearum* [18,19,20,21,22]. The final genome assembly of strain IMI 349063 contains 28 unitigs (chromosome 7 is represented by 13 small unitigs and the mitochondrial genome is represented by 3 unitigs) with a total length of 50.82 Mb. Based on the new gene annotation, a total of 14,651 protein-coding genes were predicted from the new genome assembly, 1521 fewer than the previous assembly [17].

Genome mining of *C. higginsianum* and *C. graminicola* for candidate secreted effector proteins (CSEPs), which serve as molecular weapons to evade or suppress plant immunity, revealed only 177 in *C. graminicola*, but 365 were found in *C. higginsianum* [12]. The CSEPs are mostly small, cysteine rich proteins, averaging 110 residues in *C. higginsianum*. The larger, more diversified CSEP repertoire of *C. higginsianum* might be an adaptation to evade defenses and invade a broader range of host plants. The more accurate gene annotation from the new assembly revealed many secondary metabolism (SM) key genes and putative biosynthetic pathways. Interestingly, the annotation demonstrated that *C. higginsianum* encodes one of the largest repertoires of SM key genes and SM gene clusters of any sequenced ascomycete, suggesting a large capacity to produce diverse metabolites [23,24,25,26,27,28]. Analysis of the mini-chromosomes showed that both are repeat-rich and AT-rich, gene-poor and highly enriched with genes encoding putative secreted effector proteins of unknown function, which are different in their content to the other 10 chromosome [17]. Surprisingly, a study revealed that strains lacking small chromosome 11 abort infection during biotrophy, while their ability to grow on artificial media was not affected, and that chromosome 12 can be lost without effects on virulence or growth on agar plates [29], indicating that a number of potential genes from chromosome 11 have critical functions in addressing plant host responses.

The complete genome assembly allows for analysis of genomic features including transposable elements, telomeres, structural rearrangements and large gene clusters. Moreover, this assembly can be a reference for investigations of other isolates of *C. higginsianum* or other *Colletotrichum* species, and such data should facilitate future studies including those on functional genomics in this important model phytopathogen.

### 3.2. Transcriptome Analyses

Several research studies have been carried out on transcriptomics of *C. higginsianum* associated with different developmental and infection stages. Based on flow cytometric purification, intracellular biotrophic hyphae of *C. higginsianum* from infected *Arabidopsis* leaves were purified for biotrophic stage transcriptome analysis. Six fungal genes, namely homologues of *NmrA*, saccharopine dehydrogenase, *CIH1* and three unigenes were specifically expressed in planta during the biotrophic phase, and the three unigenes (3, 125 and 143) are likely to encode small, soluble secreted proteins of unknown function that represent candidate fungal effectors [30].

RNA-Seq data for samples from infected *Arabidopsis* corresponding to pre-penetration appressoria, the early biotrophic phase and the transition to necrotrophy have been released, and the transcription levels were found to be highly dynamic [15]. At the appressorial phase, genes encoding CAZymes that were predicted to degrade cutin, cellulose hemicellulose and pectin were upregulated, which may contribute to initial host penetration, together with a larger set of enzymes that potentially remodel the fungal cell wall. During early infection, the transcriptome was dominated by secondary metabolism genes, with the majority of expressed SM gene clusters being induced before penetration and during biotrophy, and not in vitro. Furthermore, the majority of CSEP-encoding genes were strongly induced during biotrophy, implying that effector production was especially prominent during the biotrophic stage. During the switch to nectrophy, there was induction of a wide variety of lytic enzymes, presumably as the fungus feeds on moribund and necrotic tissues to allow prolific growth and colonization leading to increased spore production. Among the lytic enzymes produced, there were CAZymes and putative secreted proteases that may cut the various types of polysaccharides associated with the host cell walls. Furthermore, there was induction of many genes encoding plasma membrane transporters which may be required for movement and assimilation of metabolic products of this degradative enzymatic activity such as sugars, oligopeptides, and amino acids [15].

### 3.3. Genetic Transformation

A high efficiency transformation system using *Agrobacterium tumefaciens*-mediated transformation (ATMT) is available for *C. higginsianum* [31]. It has become established as the method of random insertional mutagenesis and targeted gene disruption using homologous recombination.

Random insertional mutagenesis is a powerful approach for discovering novel pathogenicity genes in fungi. Based on the first application of ATMT for insertional mutagenesis of *C. higginsianum*, a T-DNA insertion library was generated [31]. By using a high-throughput infection assay on *A. thaliana* seedlings, among 8850 mutants, 40 mutants showed reproducible pathogenicity defects on *Arabidopsis* and *Brassica* plants, 6 were impaired in appressorial melanization, 15 had reduced penetration ability, 14 induced host papillae or hypersensitive cell death, and 5 were affected in the transition from biotrophy to necrotrophy [31]. Flanking sequence analysis of the tagged genes led to the isolation of 14 putative pathogenicity genes (Table 1). Similarly, another T-DNA insertion library of *C. higginsianum* was also generated containing 5012 ATMT mutants, and six virulence-deficient mutants were acquired (Table 1). Identification and analysis of the T-DNA tagged loci of these mutants revealed several potential genes possibly related to virulence factors such as genes encoding a copper amine oxidase, an exosome component EXOSC1/CSL4, and other hypothetical proteins [32]. Korn et al. (2015), from a collection of 7200 insertion mutants, isolated 75 mutants with reduced symptoms. Among them, 19 were affected in host penetration, while 17 were affected in later stages of infection [33]. The location of T-DNA insertions of only 16 mutants could be identified by polymerase chain reaction (PCR) for further gene functional analysis (Table 1).

Among the T-DNA insertion library, two *C. higginsianum* mutants defective in the switch from biotrophy to necrotrophy showed high homology to conserved importin-β2 proteins. This class of importins is known to mediate the nuclear importation of pre-mRNA processing proteins in mammals, yeast and plants [34,35]. Importins are considered essential for the asexual/sexual development in *Trichoderma reesei* and pathogenicity in *Aspergillus nidulans*, *Phytophthora sojae* [36,37,38]. Importantly, there have been no reports that importin proteins are involved in the transition from biotrophy to necrotrophy. Otherwise, many T-DNA tagged loci of these mutants were annotated as hypothetical proteins, and these genes have the potential to be involved in novel functional genes possibly related to virulence factors. There needs to be more attention and penetrating research on these genes.

To validate that the integrated T-DNA is responsible for the observed phenotypes, complementation experiments or the generation of targeted knockout mutants are important. Since targeted gene mutagenesis through homologous recombination occurs with relatively low frequency in *C. higginsianum* [33], an efficient gene knockout protocol, which could increase homologous recombination frequency to 60–90%, was established based on the inactivated Ku70 and Ku80 components of the non-homologous end-joining (NHEJ) pathway in *C. higginsianum* [33,39]. The method of inactivated NHEJ pathway components to increase the homologous recombination frequency was previously reported in other filamentous fungi, and this method raised the transformation efficiency to 60–90% in *A. sojae* and *A. oryzae* [40] and by over 80% in *M. oryzae* [41]. Overall, these findings indicate that insertional mutagenesis by ATMT could be a valuable tool for genome-wide analysis of gene function in this important model pathogen.

## 4. Virulence Factors

Genome and transcriptome analyses of *C*. *higginsianum* infecting *A*. *thaliana* has shown that this fungus has many virulence factors. However, relatively few molecular determinants of virulence in *C. higginsianum* have been experimentally verified (Table 2).

### 4.1. Mitogen-Activated Protein (MAP) Kinase and cAMP/PKA Signaling Pathway

Adhesion to the plant surface is the first step in initiation of the infection process in many plant pathogenic fungi [49]. Following adhesion, physical signals such as those involving tissue hardness and hydrophobicity or chemical signals (cutin monomers and leaf waxes) induce germination and appressorial formation in several plant pathogenic fungi [50]. In eukaryotic cells, the transduction of a variety of extracellular signals and the regulation of different developmental processes are regulated by mitogen-activated protein (MAP) kinase pathways and cAMP/PKA signaling pathways [50,51]. Thus, some intensive studies on these two pathways have been carried out based on the infection process of *C. higginsianum*. ChSte7, encoding a MAPKK orthologue gene *Ste7* in yeast, was highly expressed in vegetative and invasive growth stages in *C. higginsianum*. Deletion of *ChSte7* resulted in significant reduction in vegetative growth, inability to form appressoria and also reduced invasive growth inside host plant tissues, which was similar for *M. oryzae*, *B. cinerea* and *Ustilago maydis* [44]. A *Fus3/Kss1* related MAPK gene in *C. higginsianum*, ChMK1, was also reported to play an important role in cell wall integrity, colony melanization, and pathogenicity on *A. thaliana* [46]. ChRgf, encoding a Ras guanine-nucleotide exchange factor protein, might be a control element of MAPK pathway (REF), and its deletion resulted in some phenotypes similar to those involving deletion of the two MAPK pathway genes: defects in vegetative growth, altered hyphal morphology, reduced conidiation, poor surface attachment and low germination on hydrophobic surfaces [45]. These results indicate that the MAPK pathway is involved in a critical conserved role to control the pathogenicity and growth of *C. higginsianum* through the extracellular signal transmission compared to other phytopathogenic fungi. Moreover, another extracellular signal transmission pathway, cAMP/PKA signaling pathway, was also studied in *C. higginsianum*. The PKA catalytic subunits ChPKA1 and adenylate cyclase ChAC deletion mutants were significantly reduced in hyphal growth rate, tolerance to cell wall inhibitors and conidiation, but had an increased tolerance to elevated temperatures and exogenous H_2_O_2_ [52]. In contrast, the ChPKA2 mutant had no detectable alteration of phenotypes, suggesting that ChPKA1 contributes mainly to PKA activities in *C. higginsianum* [52]. These findings suggest that the cAMP/PKA signaling pathway also contributes to growth, conidial formation, stress tolerance and pathogenesis in *C. higginsianum*.

The MAPK and cAMP/PKA signaling pathways are well known in regulation of appressorial morphogenesis and plant infection in *M. oryzae*, *C. truncatum* and some other phytopathogenic fungi [53,54,55,56], but investigation of these two signaling pathways in *C. higginsianum* still helped to provide insights into the mechanism of the *C. higginsianum*–cruciferous crop interaction, and to facilitate investigation of efficient management of anthracnose disease. Functional comparisons of MAPK and cAMP/PKA signaling pathways in *C. higginsianum* with other phytopathogenic fungi may provide a deeper understanding of pathogenic mechanisms of this fungus.

After adhesion to plant surfaces, the infection strategy of *C. higginsianum* includes two phases: an initial biotrophic phase and a subsequent necrotrophic growth phase [57]. For biotrophic growth and transition to necrotrophic growth, fine-tuned regulation of cell wall developmental processes are essential. For fungi, management of these processes involves many pathways, including kinases and the co-activator Mob-family proteins [58]. These pathways have been called morphogenesis-related [59] and the septum initiation network [60] in *Saccharomyces pombe*, or regulation of Ace2p activity and morphogenesis [61] and the mitotic exit network [62] both in *S. cerevisiae*. In this latter fungus, Cbk1 is the terminal kinase in the RAM pathway, and it is classified in the kinase subfamily of nuclear Dbf2-related or large tumor suppressor. The function of Mob-family proteins which are essential for the activation of NDR kinases was also studied in *C. higginsianum* [48]*.* The results showed that the *C. higginsianum* genome encodes three members of the Mob1/phocein protein family. ChMob1 is required for conidiation, cytokinesis and plant infection. ChMob2 binds to the conserved NDR/LATS kinase ChCbk1, and is involved in virulence on *A. thaliana* and is required for both conidiation and formation of functional appressoria. ChMob3 knockout mutants have no obvious phenotype in vegetative cells or during infection. Moreover, Mob2 and Cbk1 co-localize to the cytoplasm and are excluded from nuclei in conidia and during appressorial formation in vitro. Mutants in the two potential Mob2/Cbk1 complex targets ChSSD1 and ChACE2 genes show defects in pathogenicity.

### 4.2. Nutrition, Transporter and Amino Acid Biosynthesis

Several studies provide evidence that nutritional requirements, such as amino acid biosynthesis or nutrient availabilities, are important for fungal infection cycles and pathogenicity in many fungi [63,64,65,66]. In *C. higginsianum*, two arginine auxotroph mutants showed reduced penetration and invasive growth ability, which was restored when L-arginine was supplied. Thus arginine biosynthesis was shown to be dispensable for conidial germination and appressorial morphogenesis of *C. higginsianum*, suggesting that arginine reserves in conidia are sufficient for the completion of pre-penetration development. However, arginine biosynthesis was critical for initial host penetration by appressoria and early biotrophic growth inside living host cells [42]. Although not many genes related to nutritional requirements have been genetically analyzed in *C. higginsianum* so far, there are many such studies in other species of *Colletotrichum*. The GATA transcription factor, AreA, regulates the use of poor or complex nitrogen sources, and restricts their use when sufficient nitrogen sources are available within the organism. The AreA plays a critical role in fungal development, conidial production, and regulation of nitrogen metabolism and virulence in *C. gloeosporioides* [67]. Kre5 and Kre6 are the key enzymes in β-1,6-glucan synthesis and formation of branch points of the β-glucan network. In *C. graminicola*, RNAi-mediated reduction of KRE5 and KRE6 transcript abundance caused appressoria to burst and necrotrophic hyphae to swell, indicating that β-1,6-glucosidic bonds are essential in these cells [68]. The homologous proteins in *C. higginsianum* should play important roles in fungal development or virulence.

The ATP-binding cassette (ABC) and major facilitator superfamily (MFS) of transporters are two families that play important roles in transport processes. In recent research, MFS transporters are usually demonstrated to be involved in multidrug resistance in fungi [69]. MFS transporters are capable of transporting small molecules in response to ion gradients or function as drug:H^+^ antiporters in microorganisms. Mounting evidence indicates that MFS transporters may also indirectly control membrane potential by changing membrane lipid homeostasis and regulating internal pH and the stress response machinery and pathogenicity in fungi [70,71,72]. Moreover, some MFS transporters also are involved in secretion of phytotoxins [73,74,75,76]. Recently, a virulence-deficient mutant, Ch-1-T513, from a T-DNA insertion mutant library in *C. higginsianum* was found to have abnormal hyphae, which might be a key factor affecting virulence of the fungus. The study demonstrated that a MFS transporter named ChMfs1 is responsible for the mutant Ch-1-T513 phenotype, and ChMfs1 in *C. higginsianum* is the first reported to be involved in pathogenicity and the production of intra-hyphal hyphae [47].

The plasma membrane H^+^-ATPase is a proton pump that plays important energetic and regulatory roles in the physiology of plants and fungi controlling essential functions including nutrient uptake and intracellular pH regulation [77]. In fungal cells, the activity of the proton pump is regulated by a large number of environmental factors at both transcriptional and post-translational levels [78]. Structure and function of plasma membrane H^+^-ATPases have been extensively explored in fungi, revealing their role in vegetative growth, nutrient transport and pathogenicity [79,80,81,82,83]. In *C. higginsianum*, a potential plasma membrane H^+^-ATPase Pma2 was frequently targeted in five independent insertion mutants from the T-DNA insertion mutant library. Chpma2 deletion mutants form fully melanized appressoria but entirely fail to penetrate the host tissue. Targeted gene knockout of another plasma membrane pump gene, *ChPMA1*, gave a non-viable phenotype, indicating that *ChPMA1* may be an essential gene and encode the major H^+^-transporting ATPase [33].

### 4.3. Effectors

In the case of plant pathogenic hemibiotrophs, colonization and the initial biotrophic interaction with host cells is facilitated by pathogen-encoded small, secreted proteins termed effectors [84]. Biotrophy-specific hyphal cells play important roles in transporting effectors into the host cells and in obtaining nutrition from the host [85]. Depending on the fungal species, the interfacial matrix is either continuous with the host plant apoplast such as for Puccinales or separated into an interfacial apoplastic compartment like that in *M. oryzae*. The interfacial membrane among a variety of pathosystems ranges from undifferentiated plant plasma membranes to highly specialized membranes with complicated elaborations and unique components [86]. Vesicles are abundant in both host and pathogen cytoplasm near the interface, implying that both are involved in active production of secreted compounds into and across interfacial zones [87,88]. Shimdada et al. found some evidence for localized specialization of the interfacial membrane around BH of *C. higginsianum* [5]. Biotrophic infection by *C. higginsianum* has differences from other biotrophic fungus, and the process of interfacial membrane exchange with the fungus deserves further study.

In *C. higginsianum*, inventories of putative effectors have been predicted from the annotated genomes of *C. higginsianum* revealing 18 genes, of which six were not predicted to be secreted, and two were chitinases, leaving 10 putative secreted LysM effectors [89]. The LysM domain comprises 40–60 amino acid residues and mediates binding to chitin and peptidoglycans [89]. Chitin is a microbe-associated molecular pattern (MAMP) that can be detected by plant pattern recognition receptors (PRRs) to activate a variety of MAMP-triggered immune responses [90]. To avoid recognition by host receptors, several ascomycetes are known to produce effector proteins which either block the activity of host plant chitinases or compete with host plant receptors which bind chitin fragments [91,92,93,94,95]. The function of two effectors, ChELP1 and ChELP2, homologs of LysM proteins were characterized in *C. higginsianum*. ChELP2 has been found to be located on the surface of bulbous biotrophic hyphae at the interface with living plant cells, but it has not been discovered in necrotrophic hyphae. In previous experiments, recombinant ChELP1 and ChELP2 were found to bind chitin oligomers in vitro with high specifity and high affinity. Both proteins suppressed chitin-triggered activation of two immunity-related plant mitogen-activated protein kinases in *Arabidopsis*. These results suggested a double role for these LysM proteins as effectors for suppressing chitin-triggered immunity and as proteins essential for appressorial development and function [43].

The role of secreted effector proteins during infection by hemibiotrophic plant pathogens is poorly understood. Based on deep transcriptome sequencing and computational mining of Expressed Sequence Tags from precise infection stages, a large of planta-expressed effector candidates were found in *C. higginsianum.* Most biotrophy-associated *ChEC* genes were dramatically upregulated exclusively in planta and distinct sets of effectors are deployed in successive waves by particular fungal cell types [96]. With fluorescent protein tagging and transmission electron microscopy-immunogold labelling, early expressed effector proteins are observed to be focally secreted from appressorial penetration pores before host invasion (Figure 1A). In addition, later-expressed effectors accumulate in structures formed at the interface between primary hyphae and living host cells (Figure 1B), implicating these hyphae in effector delivery. Furthermore, the coordinated expression and secretion of antagonistic biotrophy effectors and toxin effectors contribute to fungal virulence and the regulation of hemibiotrophy in *C. higginsianum*. These findings indicate new functions for fungal infection structures that have not been reported previously, specifically the localized release of effector proteins at the interface between fungal pathogen and plant host, and associated with the penetration pore. This provides the basis to model the switch to necrotrophy from biotrophy by this fungus [96]. Future research should attempt to decipher the nature of the plant signals inducing effector gene expression and the way that they are sensed by the pathogen.

Recently, 61 putative effector proteins were separately cloned into a plant expression vector providing an N-terminal GFP tag, and the tagged proteins were transiently expressed directly inside plant cells using ATMT [97]. Among them, subcellular localization of 16 candidate effectors was verified, nine were imported into plant nuclei, three were imported into the matrix of peroxisomes, three decorated cortical microtubule arrays, and one was associated with Golgi stacks [97]. These findings revealed that plant peroxisomes, microtubules and Golgi are novel targets for fungal effectors.

## 5. Molecular Interactions

Plants usually defend against microbial pathogens by activating both localized and systemic resistance responses. These responses include hypersensitive response [98], cell-wall fortification [99], synthesis of phytoalexins [100] and production of other antimicrobial secondary metabolites or pathogenesis-related proteins (PR proteins) [101]. Signaling molecules implicated in these inducible defense systems include salicylic acid (SA), jasmonic acid (JA), ethylene (ET), abscisic acid (ABA), auxin, gibberellins (GAs), cytokinins (CKs), brassinosteroids (BRs), and reactive oxygen species (ROS) [102,103]. These phytohormones can induce defense responses individually, and also interact synergistically or antagonistically to further orchestrate downstream signaling.

A large number of fungal and oomycete pathogens have been reported to infect the model plant *A. thaliana*, either naturally or in the laboratory. As a typical hemibiotrophic fungus, *C. higginsianum* develops a series of specialized infection structures. In particular, the intracellular BH of *C. higginsianum* are equivalent to haustoria, and hence this pathosystem can provide insights into the molecular basis of biotrophy in obligately parasitic organisms, such as rusts, powdery mildews, and downy mildews, all of which are not readily culturable or genetically manipulated.

### 5.1. Primary Metabolic Pathways

For successful establishment in host plants, biotrophic and hemibiotrophic fungi need to obtain nutrients from living host cells, and effectively evade the host defense system. Colonization by a fungal pathogen is associated with multiple metabolic changes in the plant host, notably increases in the expression of several genes involved in primary metabolic pathways, synthesis or degradation of carbohydrates, amino acids, lipids, and mineral transport [104]. It has been suggested that the role of primary metabolism during plant–pathogen interactions is to support cellular energy requirements for plant defense responses [105]. The mutation of *lht1* (lysinehistidine transporter 1) in *Arabidopsis* can significantly reduce contents of glutamine, alanine, and proline, resulting in enhanced resistance not only to *C. higginsianum* but also to diverse bacterial and oomycete pathogens [106]. After inoculation with these pathogens, the *lht1* mutant also exhibited increased callose deposition, higher accumulation of SA and constitutive expression of PR-1.

Furthermore, more evidence has suggested that components of primary metabolism also can act as signals regulating various aspects of plant defense. For example, fatty acids and lipids play important roles in plant defense and and biosynthesis of the major defense hormone JA [107]. Little is known about the role of plant primary metabolism in defense against attack by this hemibiotroph. To date, several metabolic functions have been identified that influence compatibility of *C. higginsianum* with the plant host. Glycerol-3-phosphate (G3P) is an important component in carbohydrate and lipid metabolic processes. Infection of *Arabidopsis* by *C. higginsianum* leads to an increase in G3P levels and a simultaneous decrease in glycerol levels in the plant. Cells impaired in the utilization of G3P accumulated higher levels of pathogen-induced G3P, and exhibited enhanced resistance [108]. The NADP-malic enzyme catalyses the oxidative decarboxylation of L-malate using NADP+ as coenzyme, producing pyruvate, CO_2_, and NADPH is present as a multigene family [109]. In *A. thaliana*, loss of cytosolic NADP-ME2 leads to increased susceptibility to infection by pathogens such as *C. higginsianum.* The data suggest that NADP-ME2 has a function during the basal defence response, where it may be required for ROS production after pathogen recognition [110]. Since primary metabolism is essential for survival, associated genes are unlikely to be to be eliminated during natural selection, in contrast to most R-genes which are only periodically important for survival (i.e., when pathogens attack). Therefore, engineering resistance against pathogens by selection of resistance-related genes that also have a primary metabolic function is expected to provide more durable resistance.

### 5.2. Phytohormones

Genetic studies with *Arabidopsis-*signaling mutants have shown that SA-dependent responses are deployed against biotrophic pathogens, whereas ethylene- or JA-dependent responses are more important for induced resistance to necrotrophic pathogens [111], suggesting that resistance to hemibiotrophic pathogens such as *Colletotrichum* may require a combination of these pathways [112]. Genome-wide studies using cDNA arrays in *Arabidopsis* infected with the *C. higginsianum* revealed that defense reactions activate the SA-dependent signaling pathway at the early stage of the interaction between *Arabidopsis* and *C. higginsianum*, and the subsequent defense reaction may depend on the JA-dependent signaling pathway because the correlation with SA signaling decreased rapidly and that of JA-signaling increased relatively [4]. Differential defense signalling crosstalk and PR gene expression are involved in cultivar-specific resistance of kimchi cabbage plants to anthracnose, black spot and black rot diseases, and the resistance is strongly associated with the hormone-dependent transcriptional induction of defence genes [113].

### 5.3. Resistance Genes

Molecular and biochemical bases of cultivar resistance to *Colletotrichum* spp. have been investigated using genetically diverse materials [114,115,116]. Innate disease resistance responses in plants are triggered by a dual surveillance system composed of nucleotide binding-leucine rich repeat (NB-LRR) proteins encoded by resistance genes and pattern recognition receptors (PRRs) [117]. The two layers are often called MAMPs-triggered immunity (MTI) and effector triggered immunity (ETI) [118].

By a combination of quantitative trait loci (QTL) and Mendelian mapping, a single putative R locus RCH1 was identified, at the tip of chromosome 4, in the resistant *A. thaliana* ecotype Eil-0 against *C. higginsianum* [119]. By using map-based cloning and natural variation analysis of 19 *Arabidopsis* ecotypes, another dominant resistance locus RCH2 was identified against *C. higginsianum.* The locus RCH2 maps to an extensive cluster of disease-resistance loci known as MRC-J in the *Arabidopsis* ecotype Ws-0. These indicate that *Arabidopsis* resistance to *C. higginsianum* is controlled by a gene-for-gene interaction.

In *A. thaliana*, NB-LRR-type resistance (R) genes to *Pseudomonas syringae* 4 (RPS4) and to *Ralstonia solanacearum* 1 (RRS1-R) were reported to also confer resistance to *C. higginsianum* [120,121]. RRS1-R and RPS4 were also found as a complex that could help detect effectors which target WRKY proteins [122,123]. Therefore, effectors in *C. higginsianum* that target WRKY proteins may be more likely to act as *Avr* genes.

## 6. Future Perspectives

This review provides an overview of recent significant studies on the pathogenesis of *C. higginsianum* and resistance mechanisms of *Arabidopsis* against this hemibiotrophic fungus. However, there are many issues worth investigating.

Firstly, the genome sequence of *C. higginsianum* has revealed a large number of putative effector proteins, but few effector proteins have been experimentally confirmed or characterized to date. The identification of virulence targets for the hundreds of candidate effectors predicted from genome sequencing remains a major challenge, partly because protocols for high-throughput plant cellular assays are lacking. Thus, establishing a reliable high-efficiency protocol for screening effectors of *C. higginsianum* would facilitate future functional identification in this important model pathogen. Moreover, previous studies showed that two effector proteins, ChELP1 and ChELP2, at the biotrophic stage in planta may be critical for suppressing chitin-triggered immune responses, while the basal expression levels in appressoria in vitro and in planta are required for efficient substrate penetration. Further work is needed to elucidate how these proteins contribute to appressorial function and the switch to invasive hyphal growth. The complete genome sequence of *C. higginsianum* revealed that chromosomes 11 and 12 are also enriched in genes encoding potential effector proteins which differ from the core genome. Lack of chromosome 11 leads to aborted infection during biotrophy, indicating that a number of potential genes from chromosome 11 have critical functions in manipulating plant host responses, and these can be selectively analyzed in future work to evaluate their possible function.

Secondly, comparative analyses of interactions between *Arabidopsis* and both non-adapted and adapted *Colletotrichum* species revealed that the adapted pathogen *C. higginsianum* induced papillary callose at a much lower frequency than non-adapted *Colletotrichum* species, indicating that this fungus may suppress pre-penetration resistance at the cell periphery [5]. The mechanism of *C. higginsianum* suppression of pre-penetration resistance remains to be elucidated by future experiments.

Finally, many genes for synthesis of secondary metabolites are up-regulated during plant infection, and many genes are specifically expressed during the biotrophic stage in *C. higginsianum*, indicating that appropriate gene expression during the biotrophic stage is a key for successful establishment of this fungus in host plants. Thus, the *C. higginsianum-Arabidopsis* pathosystem has tremendous potential for discovery of novel bioactive molecules, and identification of the corresponding biosynthetic pathways.

## Figures and Tables

**Table 1:** Summary of *Colletotrichum higginsianum* genes identified from T-DNA flanking sequences.

Mutant	Insertion ^a^	T-DNA Insertion ^b^	Putative Function (NCBI Accession) ^c^	Reference
path-5	1	In predicted open reading frame (ORF)	Unknown	[31]
path-7	2	In ORF	Hypothetical protein (FG06146.1)	
		1.5 kb upstream	Hypothetical protein (FG06145.1)	
path-8	1	In predicted ORF	Unknown	
path-9	1	1 kb downstream	Endo–1,3(4)–β–glucanase (AFUA_1G05290)	
path-12	1	In ORF	MFS transporter (NFIA_086030)	
path-16	1	In ORF	Ornithine decarboxylase (AY602214)	
path-19	1	In ORF	Arg–6 protein (EAA35492.1)	
path-23	2	620 bp upstream	Hypothetical protein (FG02446.1)	
		In predicted ORF	Unknown	
path-29	1	730 bp upstream	ATP–binding endoribonuclease (ACLA_048430)	
path-35	1	In ORF	Carbamoyl–phosphate synthetase (EAA36214.1)	
path-36	1	620 bp upstream	Importin β2 subunit (AFUA_1G15900)	
path-38	1	In ORF	Importin β2 subunit (AFUA_1G15900)	
T732	1	168 bp downstream	Copper amine oxidase (XP_001826965)	[32]
T734	1	In ORF	Hypothetical protein (ELA33048)	
B30	2	In ORF	Exosome component EXOSC1/CSL4 (EFQ29835)	
		850 bp upstream	DUF221 domain protein (EFY94646)	
T45	Unknown		Hypothetical protein (EFQ29552)	
vir-2	2	supercontig_1.2671, 583, RB	Phosphoribosylaminoimidazole carboxylase (EFQ26499.1)	[33]
vir-10	2	contig05930, 16777, LB	Kelch domain-containing protein (EFQ26610.1)	
vir-12	2	supercontig_1.3174,1154, LB	Plasma-membrane proton-efflux P-type ATPase	
vir-14	2	supercontig_1.6150,870, RB	ABC transporter (EFQ25092.1)	
		supercontig_1.903,6335, LB	Nucleoside-diphosphate-sugar epimerase	
vir-22	1	supercontig_1.3174,1748, RB	Plasma-membrane proton-efflux P-type ATPase	
vir-24	1	supercontig_1.3174,1422, RB	Plasma-membrane proton-efflux P-type ATPase	
vir-27	2	supercontig_1.6150,873, RB	ABC transporter (EFQ25092.1)	
		supercontig_1.826,7944, RB	STE like transcription factor	
vir-51	1	supercontig_1.1848,6585, LB	Unknown	
vir-52	2	contig 00557	Alanine dehydrogenase/PNT domain containing protein (EFQ25467.1)	
		contig 11896	FAD dependent oxidoreductase superfamily protein (XP_007280006)	
vir-53	2	supercontig_1.6692, RB	Unknown	
vir-56	3	supercontig_1.66,3878, LB	Peroxisomal membrane protein 24	
vir-76	2	supercontig_1.56,17248, LB	Spindle assembly checkpoint component MAD1	
vir-84	2	supercontig_1.3742,1175, LB	Sporulation protein RMD1 (ELA35952.1)	
vir-88	2	supercontig_1.5277,868–879	Mob1/phocein family protein (EFQ26211.1)	
vir-97	2	supercontig_1.3174,812, LB	Plasma-membrane proton-efflux P-type ATPase	
vir-102	1	supercontig_1.3174,793, LB	Plasma-membrane proton-efflux P-type ATPase	

^a^ Number of insertion sites determined by Southern Blot analysis. ^b^ Locations of T-DNA insertion sites or position of the T-DNA border sequence in the *Colletotrichum* database Supercontigs. Sequence names are shown with left border (LB) or right border (RB). ^c^ Open reading frames (ORFs) predicted by Softberry were used in the BLAST search.

**Table 2:** Genes involved in virulence have been reported in *Colletotrichum higginsianum*.

Gene	ID	Description	Reference
*path-19*	CH063_11554	Putative Arg6 precursor	[42]
*path-35*	CH063_15109	Carbamoyl–phosphate synthetase	[42]
*Ch-MEL1*	unknown	Hypothetical protein	[32]
*ChPma2*	CH063_09060	Plasma-membrane proton-efflux P-type ATPase	[33]
*ChELP1*	CH063_13023	LysM effectors	[43]
*ChELP2*	CH063_04445	LysM effectors	[43]
*ChSte7*	CH063 02455	Serine/threonine protein kinases	[44]
*ChRgf*	CH063_04363	Ras guanine-nucleotide exchange factor	[45]
*ChMK1*	CH063_08490	Fus3/Kss1-relatedMAPKgene	[46]
*ChMfs1*	CH063_12120	Major facilitator superfamily (MFS) transporter	[47]
*ChMob2*	CH063_12012	Mob1/phocein family protein	[48]
*ChCbk1*	CH063_12968	NDR/LATS kinase	[48]
